# Levosimendan Form I, C_14_H_12_N_6_O

**DOI:** 10.1107/S2056989026005190

**Published:** 2026-05-22

**Authors:** Jacob K. Salazar, James A. Kaduk, Anja Dosen, Thomas N. Blanton

**Affiliations:** a North Central College, Department of Chemistry 131 S Loomis St Naperville IL 60540 USA; b North Central College, Department of Physics 131 S Loomis St Naperville IL 60540 USA; c ICDD, 12 Campus Blvd., Newtown Square PA 19073-3273, USA; Universidad de Los Andes, Venezuela

**Keywords:** powder diffraction, Simdax®, levosimendan, Rietveld refinement, density functional theory

## Abstract

The crystal structure of levosimendan Form I [systematic name: 2-({4-[(4*R*)-4-methyl-6-oxo-4,5-di­hydro-1*H*-pyridazin-3-yl]phen­yl}hydrazinyl­idene)propanedi­nitrile], C_14_H_12_N_6_O, has been solved and refined using synchrotron X-ray powder diffraction data, and optimized using density functional theory techniques. Levosimendan Form I crystallizes in space group *P*2_1_2_1_2_1_ (#19). The crystal structure is characterized by nearly parallel stacking of the mol­ecules parallel to the *bc* plane. N—H⋯O and N—H⋯N hydrogen bonds link the mol­ecules within the layers, with graph sets *C*(11) and *C*(13) and larger patterns.

## Abstract 2

The crystal structure of levosimendan Form I has been solved and refined using synchrotron X-ray powder diffraction data, and optimized using density functional theory techniques.

## 1. Chemical context

Levosimendan (marketed as Simdax®) is used to treat congestive heart failure. It functions as a calcium sensitizer inotrope medication used to control the force of heart contractions. The systematic name (CAS Registry Number 141505-33-1) is 2-[[4-[(4*R*)-4-methyl-6-oxo-4,5-di­hydro-1*H*-pyridazin-3-yl]phen­yl]hydrazinyl­idene]propanedi­nitrile.
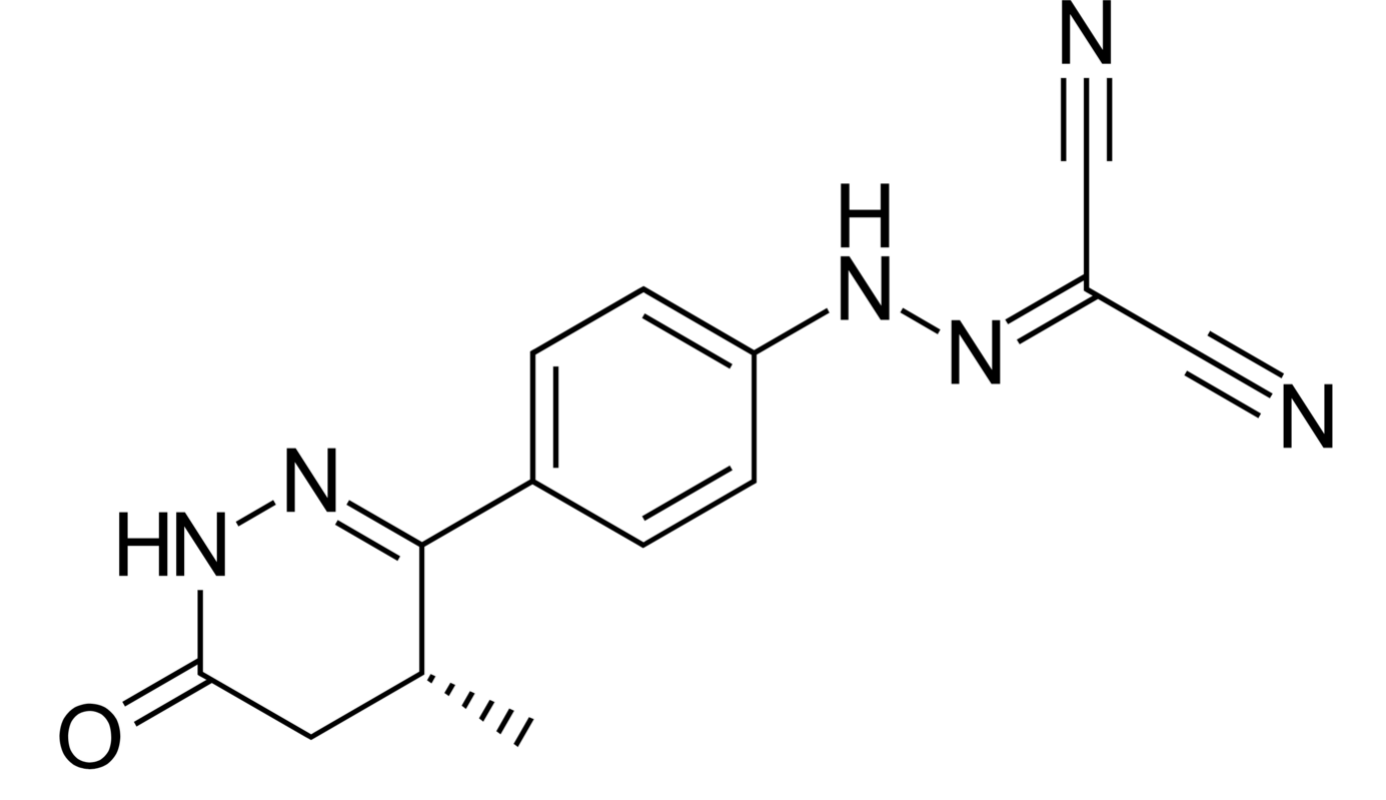


Compositions containing crystalline levosimendan Form I are claimed in US Patent 6,355,269 B1 (Backman *et al.*, 2002 ▸; Orion Corporation), and powder data for pure Form I are provided. A process for preparing levosimendan Form II is claimed in European Patent Application EP 3,424,908 A1 (Singhania, 2017 ▸; Melody Healthcare Pvt. Ltd.), and powder data are provided. However, crystal structure data are not reported.

This work was carried out as part of a project (Kaduk *et al.*, 2014 ▸) to determine the crystal structures of large-volume commercial pharmaceuticals, and include high-quality powder diffraction data for them in the Powder Diffraction File (Kabekkodu *et al.*, 2024 ▸).

## 2. Structural commentary

**Figure 1:**
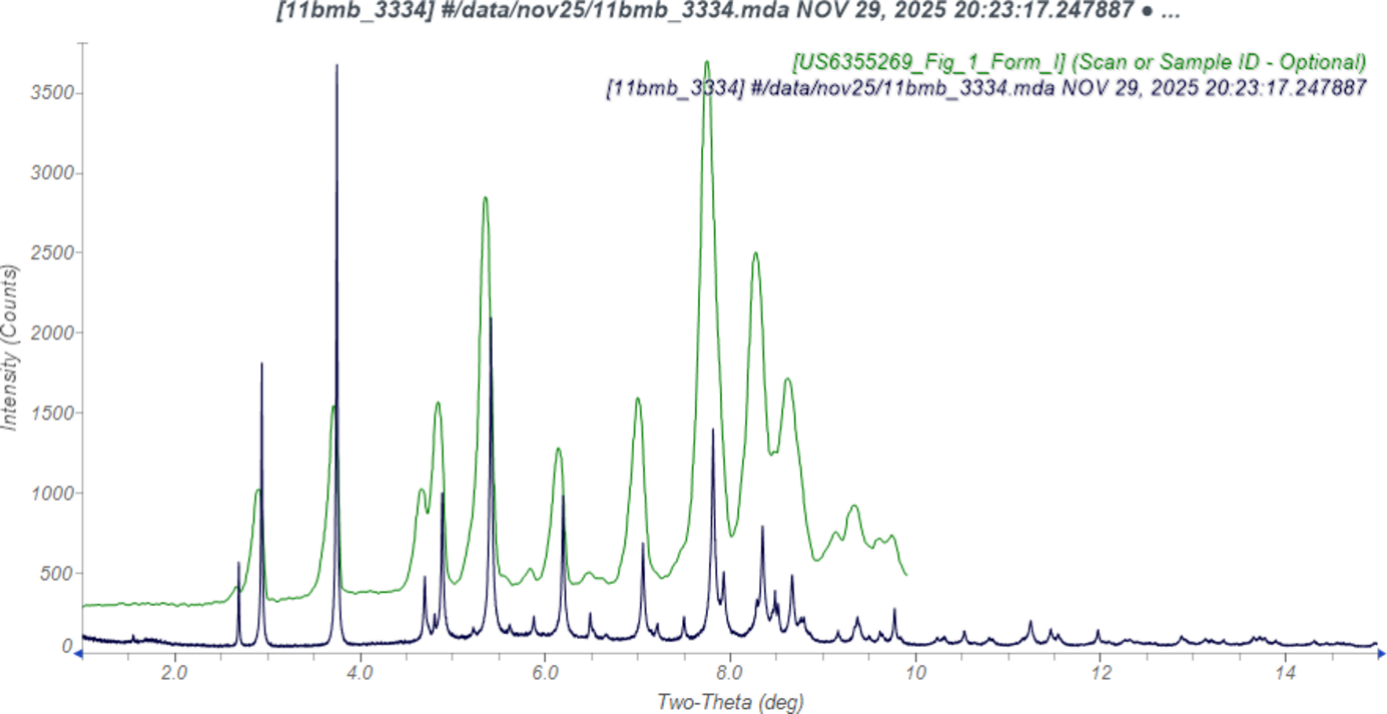
Comparison of the synchrotron pattern of levosimendan Form I (black) from this study to the laboratory XRD pattern reported by Backman *et al.* (2002 ▸; green). The patent pattern (measured using Cu *K*α radiation) was digitized using *UN-SCAN-IT* (Silk Scientific, 2013 ▸) and converted to the synchrotron wavelength of 0.4687342 Å using *JADE Pro* (MDI, 2025 ▸). Image generated using *JADE Pro* (MDI, 2025 ▸).

The synchrotron pattern of levosimendan is similar enough to that reported by Backman *et al.* (2002 ▸) for Form I (Fig. 1 ▸) to conclude that they represent the same material. The patent pattern exhibits significant displacement/transparency peak position errors, as well as preferred orientation.

**Figure 2:**
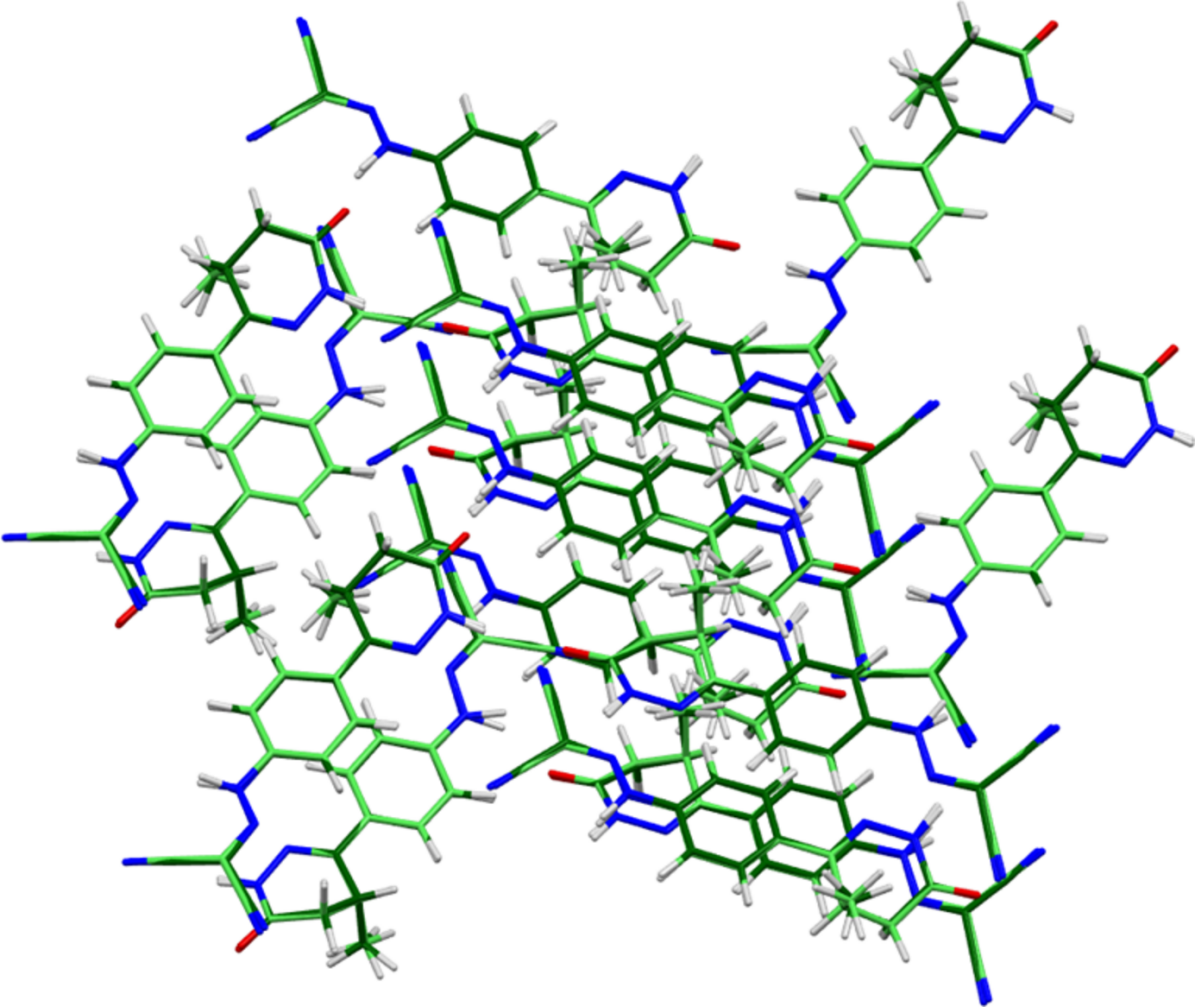
Comparison of the Rietveld-refined (colored by atom type) and *VASP*-optimized (pale green) structures of levosimendan Form I, calculated using the *Mercury* CSD-Materials/Search/Crystal Packing Similarity tool. The root-mean-square Cartesian displacement is 0.093 Å. Image generated using *Mercury* (Macrae *et al.*, 2020 ▸).

**Figure 3:**
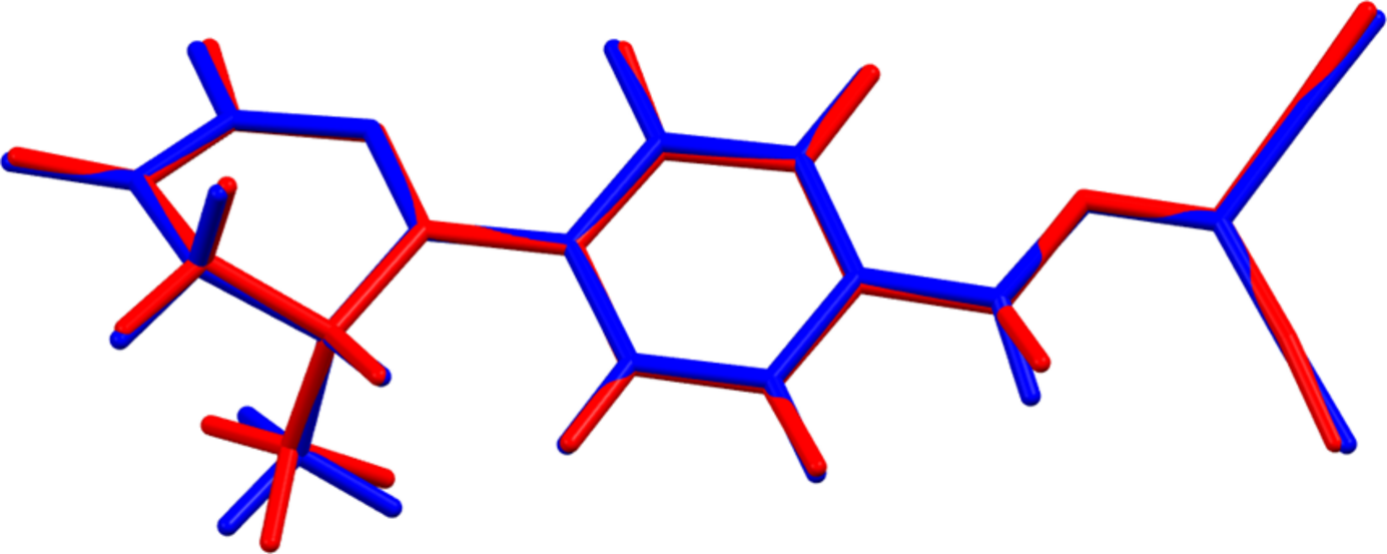
Comparison of the refined structure of levosimendan Form I (red) to the *VASP*-optimized structure (blue). The comparison was generated using the *Mercury* Calculate/Mol­ecule Overlay tool; the r.m.s. difference is 0.083 Å. Image generated using *Mercury* (Macrae *et al.*, 2020 ▸).

**Figure 4:**
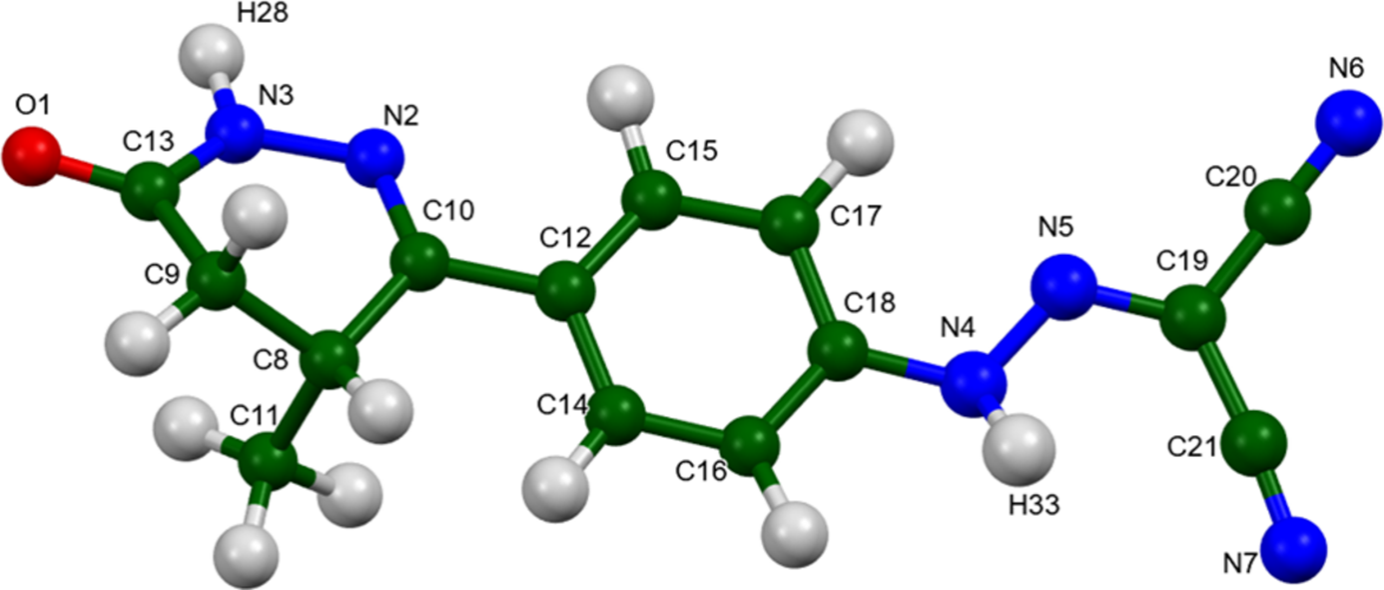
The asymmetric unit of levosimendan, with the atom numbering. The atoms are represented by 50% probability spheroids. Image generated using *Mercury* (Macrae *et al.*, 2020 ▸).

The root-mean-square difference of the non-H atoms in the Rietveld-refined and *VASP*-optimized structures of levosimendan, calculated using the *Mercury* CSD-Materials/Search/Crystal Packing Similarity tool (Macrae *et al.*, 2020 ▸) is 0.093 Å (Fig. 2 ▸); the structures are essentially identical. The root-mean-square Cartesian displacement of the non-H atoms in the refined and optimized structures, calculated using the *Mercury* Calculate/Mol­ecule Overlay tool, is 0.083 Å (Fig. 3 ▸); the maximum difference is 0.167 Å, at N6. The agreements are within the normal range for correct structures (van de Streek & Neumann, 2014 ▸). The asymmetric unit is illustrated in Fig. 4 ▸. The remaining discussion will emphasize the *VASP*-optimized structure.

All of the bond distances, bond angles, and torsion angles fall within the normal ranges indicated by a *Mercury* Mogul Geometry check (Macrae *et al.*, 2020 ▸). Quantum chemical geometry optimization of the isolated levosimendan mol­ecule (DFT/B3LYP/6-31G*/water) using *Spartan ’24* (Wavefunction, 2025 ▸) indicated that the observed conformation is 2.3 kcal mol^−1^ higher in energy than a local minimum, which has a very similar conformation. The global minimum-energy conformation is 3.3 kcal mol^−1^ lower in energy, but is folded on itself. Inter­molecular inter­actions are thus important to determine the observed solid-state conformation.

## 3. Supra­molecular features

**Figure 5:**
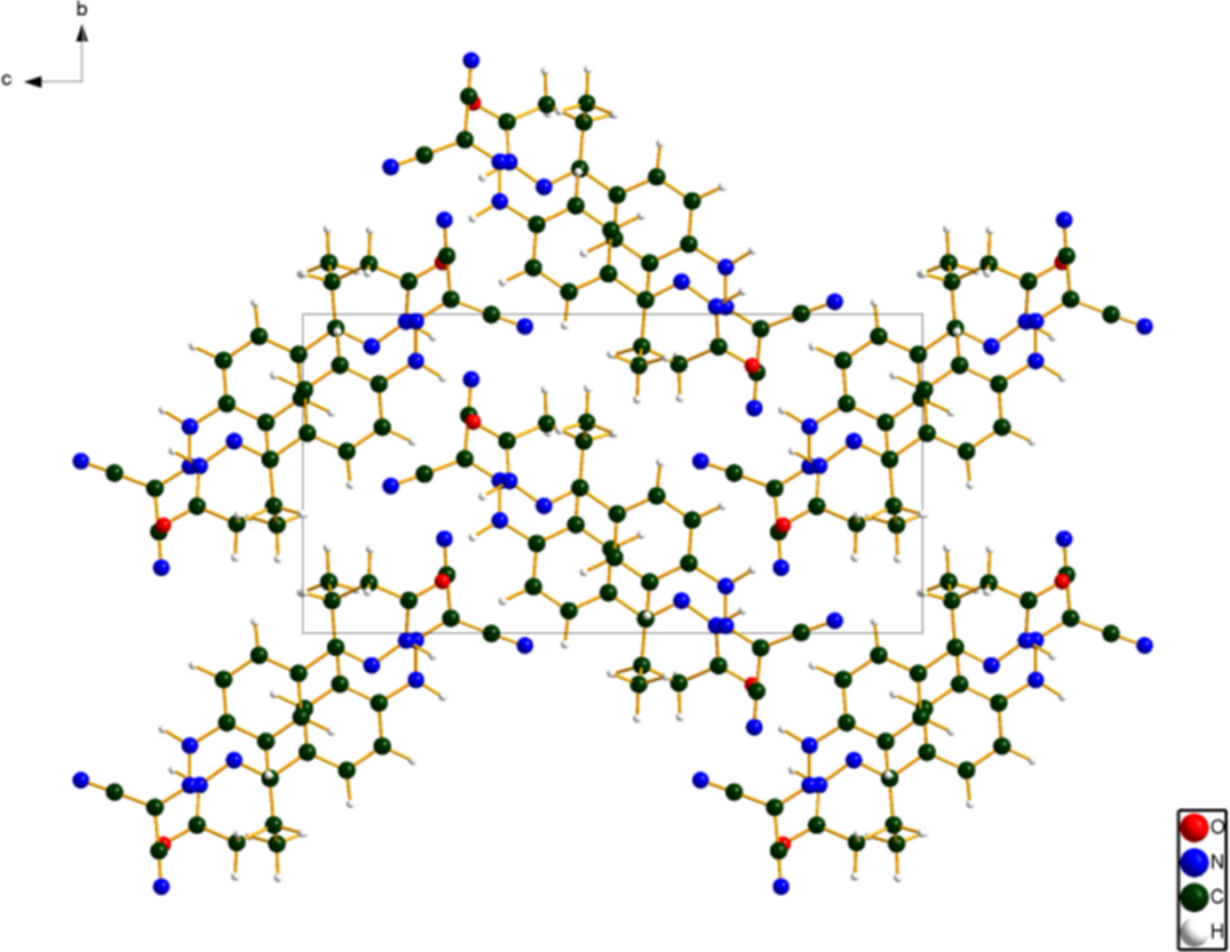
Crystal structure of levosimendan, viewed down the *a*-axis. Image generated using *DIAMOND* (Crystal Impact, 2025 ▸).

**Figure 6:**
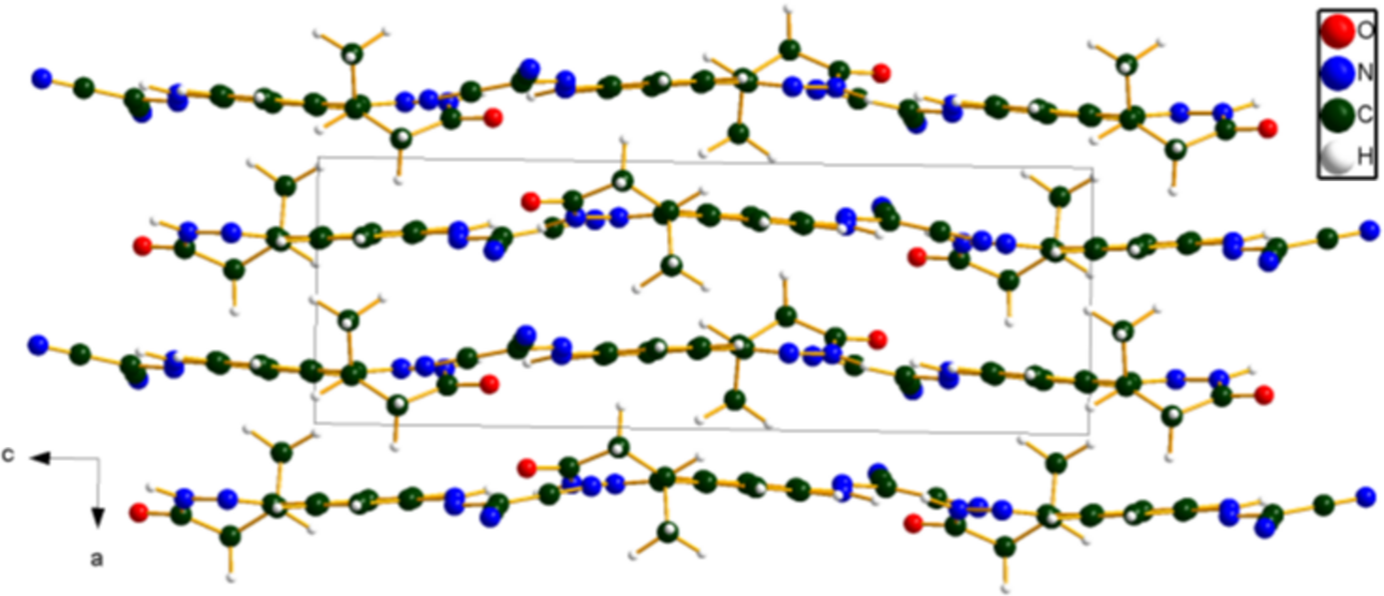
Crystal structure of levosimendan, viewed down the *b* axis. Image generated using *DIAMOND* (Crystal Impact, 2025 ▸).

A view of the crystal structure down the short *a*-axis (Fig. 5 ▸) shows the mol­ecules reasonably clearly, but obscures the nearly parallel stacking of the mol­ecules (Fig. 6 ▸) parallel to the *bc* plane. N—H⋯O and N—H⋯N hydrogen bonds (Table 1 ▸) link the mol­ecules within the layers. The mean plane of the mol­ecules is approximately (5
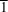

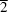
). The *Mercury* Aromatics Analyser indicates two strong (*d* = 4.42 Å) inter­actions between the phenyl rings.

Analysis of the contributions to the total crystal energy of the structure using the Forcite module of *Materials Studio* (Dassault Systèmes, 2025 ▸) indicated that bond, angle, and torsion distortion terms contribute about equally to the intra­molecular energy. The inter­molecular energy is dominated by van der Waals attractions, which in this force field based analysis include hydrogen bonds. The hydrogen bonds are better discussed using the results of the DFT calculation.

**Figure 7:**
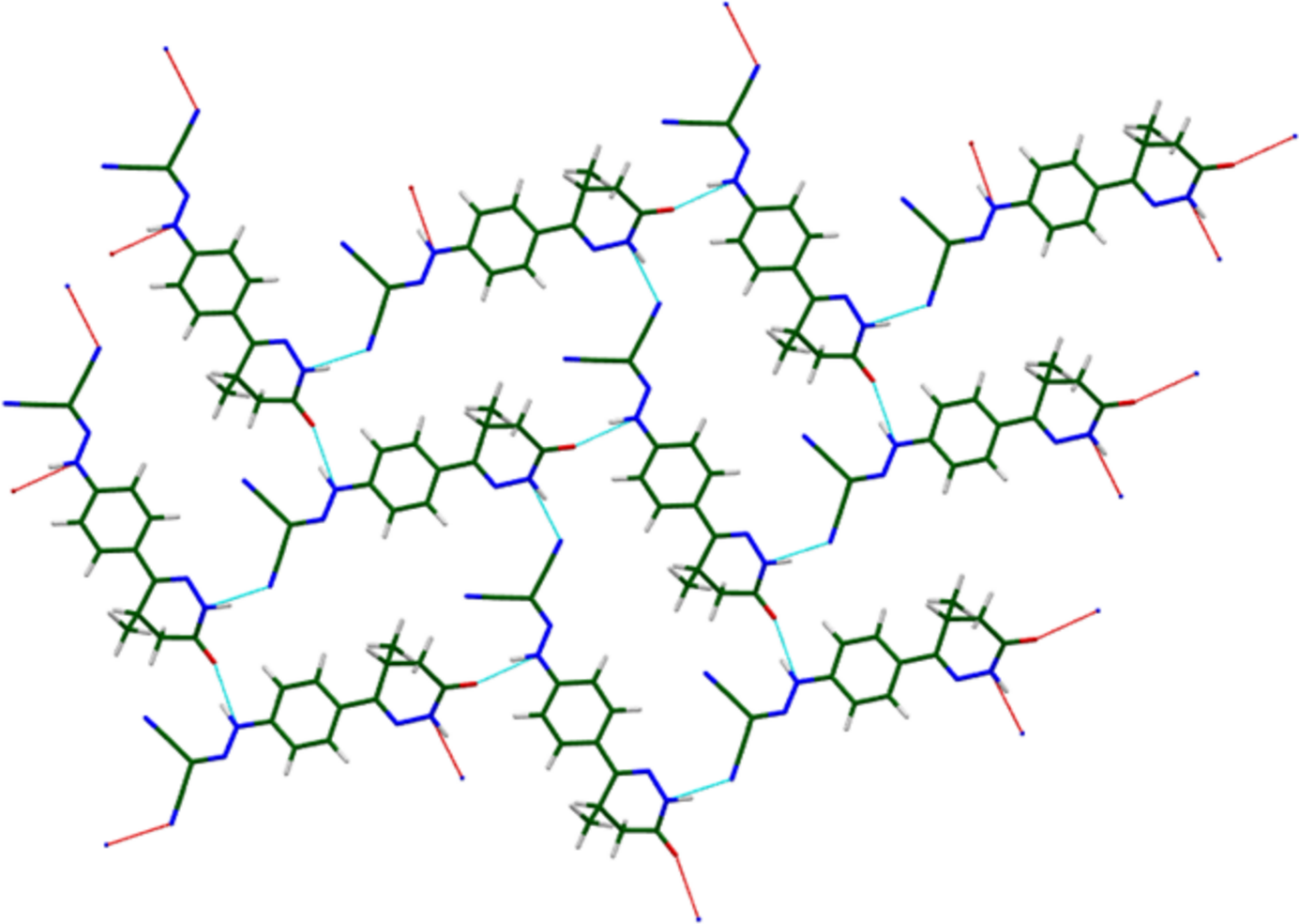
The hydrogen bonds in the *bc* plane of the layers in levosimendan Form I. Image generated using *Mercury* (Macrae *et al.*, 2020 ▸). The red dashed lines indicate hydrogen bonds generated automatically using the definitions in *Mercury*, and the cyan dashed lines indicate hydrogen bonds generated manually using the *Mercury* Expand Contacts tool.

A strong N4—H33⋯O1 hydrogen bond (Table 1 ▸) links an amino group and the carbonyl group. The energy of this hydrogen bond (5.6 kcal mol^−1^) was calculated using the correlation of Wheatley & Kaduk (2019 ▸). An N3—H28⋯N6 hydrogen bond links the other amino group with one of the cyano groups. These two classical hydrogen bonds link the mol­ecules within the layers (Fig. 7 ▸). The graph sets (Etter, 1990 ▸; Bernstein *et al.*, 1995 ▸; Motherwell *et al.*, 2000 ▸) of these two hydrogen bonds are *C*(11) and *C*(13), and they form larger patterns with graph sets 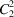
(10), 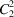
(24) and higher. Intra- and inter­molecular C—H⋯O, C—H⋯N, and C—H⋯C hydrogen bonds also contribute to the lattice energy.

**Figure 8:**
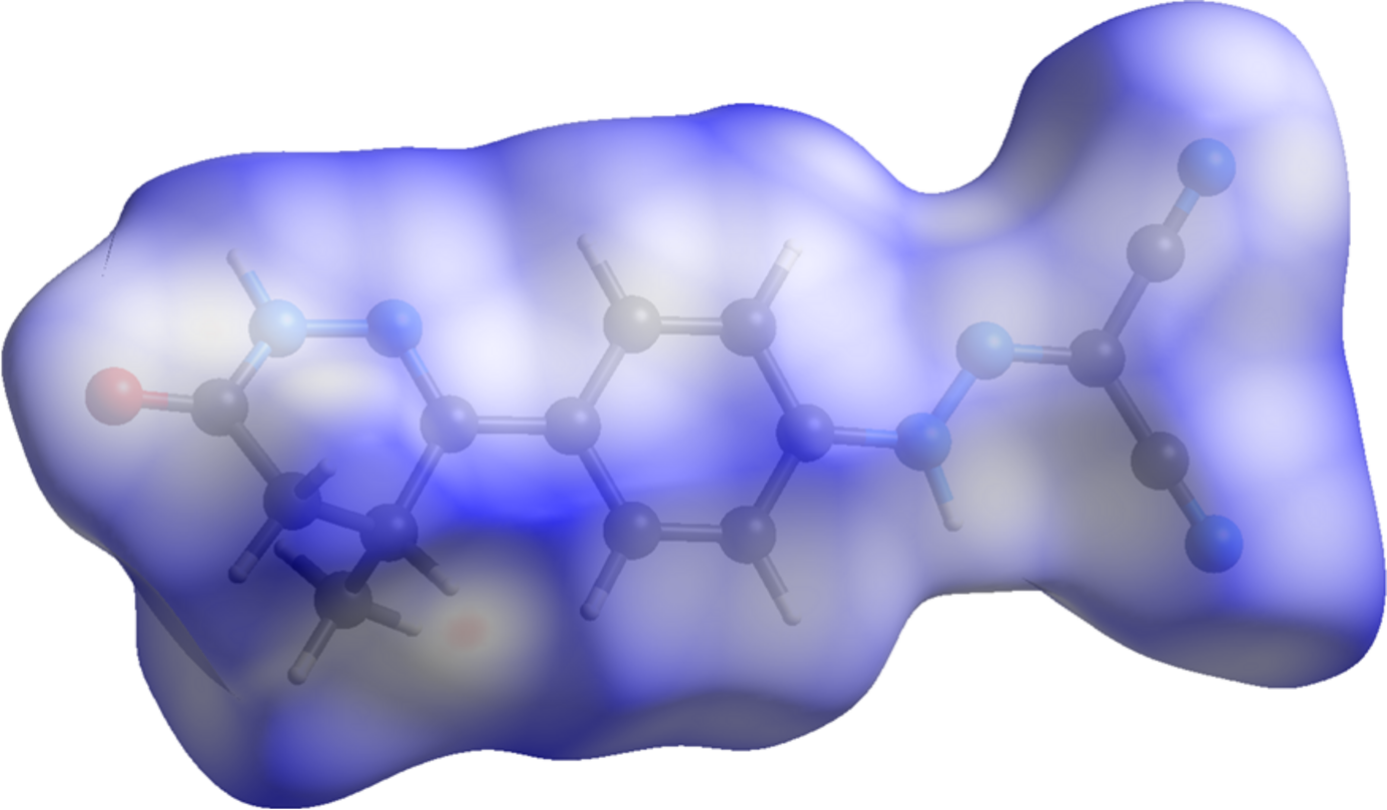
The Hirshfeld surface of levosimendan Form I. Inter­molecular contacts longer than the sums of the van der Waals radii are colored blue, and contacts shorter than the sums of the radii are colored red. Contacts equal to the sums of radii are white. Image generated using *CrystalExplorer* (Spackman *et al.*, 2021 ▸).

The volume enclosed by the Hirshfeld surface of levosimendan (Fig. 8 ▸; Hirshfeld, 1977 ▸; Spackman *et al.*, 2021 ▸) is 346.56 Å^3^, 97.93% of 1/4 of the unit-cell volume. The packing density is thus typical. Surprisingly, the hydrogen bonds are not prominent among the close contacts (red in Fig. 8 ▸). The volume/non-hydrogen atom is smaller than normal, at 16.8 Å^3^.

The Bravais–Friedel–Donnay–Harker (Bravais, 1866 ▸; Friedel, 1907 ▸; Donnay & Harker, 1937 ▸) algorithm suggests that we might expect elongated morphology for levosimendan, with [100] as the long axis. A second-order spherical harmonic model for preferred orientation was included. The texture index was 1.002, indicating that the preferred orientation was negligible in this rotated capillary specimen.

## 4. Database survey

A name search in the Powder Diffraction File (Kabekkodu *et al.*, 2024 ▸) yielded no hits. A reduced cell search in the Cambridge Structural Database (CSD Version 2026.1.0; Groom *et al.*, 2016 ▸), combined with the chemistry C, H, N, and O only, yielded 30 hits, but no structures of levosimendan or its derivatives. The powder pattern has been submitted to ICDD for inclusion in the Powder Diffraction File™ (PDF®)

## 5. Synthesis and crystallization

Levosimendan was a commercial reagent, purchased from TargetMol (Batch #120246), and was used as received.

## 6. Refinement

Crystal data, data collection and structure refinement details are summarized in Table 2 ▸. The bright-orange powder was packed into a 1.5 mm diameter Kapton capillary, and rotated during the measurement at ∼50 Hz. The powder pattern was measured at 295 K at beam line 11-BM (Lee *et al.*, 2008 ▸; Wang *et al.*, 2008 ▸; Antao *et al.*, 2008 ▸) of the Advanced Photon Source at Argonne National Laboratory using a wavelength of 0.4687342 Å from 0.5–50° in 2θ with a step size of 0.001° and a counting time of 0.1 sec/step. The high-resolution powder diffraction data were collected using twelve silicon crystal analyzers that allow for high angular resolution, high precision, and accurate peak positions. A mixture of silicon (NIST SRM 640c) and alumina (NIST SRM 676a) standards (ratio Al_2_O_3_:Si = 2:1 by weight) was used to calibrate the instrument and refine the monochromatic wavelength used in the experiment.

The pattern was indexed on a primitive ortho­rhom­bic unit cell with *a* = 6.90090, *b* = 10.33125, *c* = 20.06161 Å, *V* = 1430.29 Å^3^, and *Z* = 4 using *JADE Pro* (MDI, 2025 ▸). The space group suggested by *EXPO2014* (Altomare *et al.*, 2013 ▸) was *P*2_1_2_1_2_1_, which was confirmed by the successful solution and refinement of the structure.

The mol­ecular structure of levosimendan was downloaded from PubChem (Kim *et al.*, 2023 ▸) as Conformer3D_COMPOUND_CID_3033825.sdf. It was converted to a *.mol2 file using *Mercury* (Macrae *et al.*, 2020 ▸). The structure was solved using Monte Carlo simulated annealing techniques as implemented in *EXPO2014* (Altomare *et al.*, 2013 ▸).

Rietveld refinement was carried out using *GSAS-II* (Toby & Von Dreele, 2013 ▸). Only the 2.0–25.0° portion of the pattern was included in the refinements (*d_min_* = 1.079 Å). All non-H bond distances and angles were subjected to restraints, based on a *Mercury*/Mogul Geometry Check (Sykes *et al.*, 2011 ▸; Bruno *et al.*, 2004 ▸). The Mogul average and standard deviation for each qu­antity were used as the restraint parameters. The phenyl ring was restrained to be planar. The restraints contributed 4.4% to the overall *χ^2^*. The hydrogen atoms were included in calculated positions, which were recalculated during the refinement using *Materials Studio* (Dassault Systèmes, 2024). The *U*_iso_ of the non-H atoms were grouped by chemical similarity. The *U_iso_* of the H atoms were fixed at 1.2× the *U*_iso_ of the heavy atom to which they are attached. The peak profiles were described using the generalized microstrain model (Stephens, 1999 ▸). The background was modeled using a six-term shifted Chebyshev polynomial, with peaks at 5.71 and 8.19° to model the scattering from the Kapton capillary and any amorphous component of the sample. The background is different from the usual one from the Kapton capillary, suggesting that the sample really does contain an amorphous component. A few unindexed peaks were present in the pattern. These were best matched by PDF entry 02-072-2436 for poly(ɛ-caprolactone) (CSD Refcode WIMXAR; Bittiger *et al.*, 1970 ▸), which was included in the refinement as a second phase. Its concentration was refined to 0.3 wt%.

**Figure 9:**
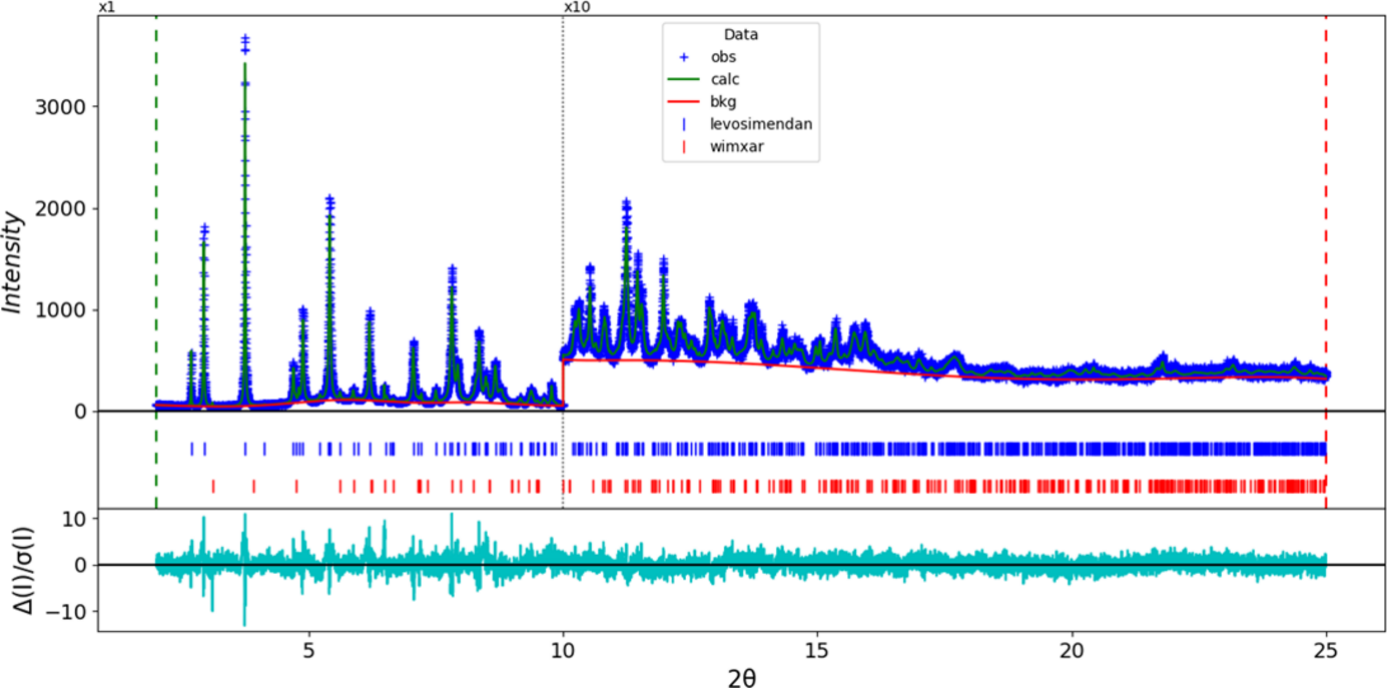
The Rietveld plot for levosimendan. The blue crosses represent the observed data points, and the green line is the calculated pattern. The cyan curve is the normalized error plot, and the red line is the background curve. The blue tick marks indicate the peak positions for levosimendan. The vertical scale has been multiplied by a factor of 10× for 2θ > 10.0°.

The final refinement of 89 variables using 23,001 observations and 52 restraints yielded the residuals *R*_wp_ = 0.06661 and GOF = 1.45. The largest peak (1.36 Å from C8) and hole (1.20 Å from C17) in the difference Fourier map were 0.13 (4) and −0.13 (4) e Å^−3^, respectively. The final Rietveld plot is shown in Fig. 9 ▸. The largest features in the normalized error plot are in the shapes of some of the strong low-angle peaks.

The crystal structure of levosimendan was optimized (fixed experimental unit cell) with density functional theory techniques using *VASP* (Kresse & Furthmüller, 1996 ▸) through the *MedeA* graphical inter­face (Materials Design, 2024 ▸). The calculation was carried out on 32 cores of a 144-core (768 Gb memory) HPE Superdome Flex 280 Linux server at North Central College. The calculation used the GGA-PBE functional, a plane wave cutoff energy of 400.0 eV, and a *k*-point spacing of 0.5 Å^−1^ leading to a 2 × 2× 1 mesh, and took ∼2.6 h. Single-point density functional theory calculations (fixed experimental cell) and population analysis were carried out using *CRYSTAL23* (Erba *et al.*, 2023 ▸); (fixed experimental cell) and population analysis were carried out using *CRYSTAL17* (Dovesi *et al.*, 2018 ▸). The basis sets for the H, C, N and O atoms in the calculation were those of Gatti *et al.* (1994 ▸). The calculations were run on a 3.5 GHz PC using 8 *k*-points and the B3LYP functional, and took ∼1.4 h.

## Supplementary Material

Crystal structure: contains datablock(s) levosimendan_publ, levosimendan_overall, levosimendan, poly_caprolactone, levosimendan_pwd_0, levosimendan_VASP. DOI: 10.1107/S2056989026005190/jw2008sup1.cif

Supporting information file. DOI: 10.1107/S2056989026005190/jw2008levosimendansup2.cml

CCDC references: 2554934, 2554935, 2554933

Additional supporting information:  crystallographic information; 3D view; checkCIF report

## Figures and Tables

**Table 1:** Hydrogen-bond geometry (Å, °)

*D*—H⋯*A*	*D*—H	H⋯*A*	*D*⋯*A*	*D*—H⋯*A*	Mulliken overlap
N4—H33⋯O1	1.045	1.826	2.813	156.1	0.058
N4—H33⋯C21	1.045	2.566	2.858	95.2	0.011
N3—H28⋯N6	1.031	1.948	2.947	162.3	0.048
C16—H31⋯O1	1.089	2.170	3.049	136.0	0.031
C15—H30⋯N2	1.090	2.502	2.821	95.3	0.014
C9—H24⋯N7	1.097	2.346	3.366	153.9	0.016

**Table 2:** Experimental details

	levosimendan
Crystal data	
Chemical formula	C_14_H_12_N_6_O
*M*_r_	280.29
Crystal system, space group	Orthorhombic, *P*2_1_2_1_2_1_
Temperature (K)	295
*a*, *b*, *c* (Å)	6.88117 (5), 10.29085 (14), 19.9896 (2)
*V* (Å^3^)	1415.53 (2)
*Z*	4
Radiation type	Synchrotron, λ = 0.46873 Å
μ (mm^−1^)	?
Specimen shape, size (mm)	Cylinder, 2 × 1.5

Data collection	
Diffractometer	11-BM, APS
Specimen mounting	Kapton capillary
Data collection mode	Transmission
Scan method	Step
2θ values (°)	2θ_min_ = 0.510, 2θ_max_ = 49.995, 2θ_step_ = 0.001

Refinement	
*R* factors and goodness of fit	*R*_p_ = 0.054, *R*_wp_ = 0.065, *R*_exp_ = 0.046, χ^2^ = 2.117
No. of parameters	89
No. of restraints	52
(Δ/σ)_max_	10.698

Computer programs: *GSAS-II* (Toby & Von Dreele, 2013 ▸).

## supplementary crystallographic information

### 2-({4-[(4R)-4-methyl-6-oxo-4,5-dihydro-1H-pyridazin-3-yl]phenyl}hydrazinylidene)propanedinitrile (levosimendan) Crystal data

**Table:**

C_14_H_12_N_6_O	*V* = 1415.53 (2) Å^3^
*M**_r_* = 280.29	*Z* = 4
Orthorhombic, *P*2_1_2_1_2_1_	*D*_x_ = 1.315 Mg m^−^^3^
*a* = 6.88117 (5) Å	Synchrotron radiation
*b* = 10.29085 (14) Å	*T* = 295 K
*c* = 19.9896 (2) Å	cylinder, 2 × 1.5 mm

### 2-({4-[(4R)-4-methyl-6-oxo-4,5-dihydro-1H-pyridazin-3-yl]phenyl}hydrazinylidene)propanedinitrile (levosimendan) Data collection

**Table:**

11-BM, APS diffractometer	Data collection mode: transmission
Specimen mounting: Kapton capillary	Scan method: step

### 2-({4-[(4R)-4-methyl-6-oxo-4,5-dihydro-1H-pyridazin-3-yl]phenyl}hydrazinylidene)propanedinitrile (levosimendan) Refinement

**Table:**

Weighting scheme based on measured s.u.'s	Preferred orientation correction: Simple spherical harmonic correction Order = 2 Coefficients: 0:0:C(2,0) = -0.0780; 0:0:C(2,2) = -0.0670

### 2-({4-[(4R)-4-methyl-6-oxo-4,5-dihydro-1H-pyridazin-3-yl]phenyl}hydrazinylidene)propanedinitrile (levosimendan) Fractional atomic coordinates and isotropic or equivalent isotropic displacement parameters (Å^2^)

**Table:**

	*x*	*y*	*z*	*U*_iso_*/*U*_eq_	
O1	0.3588 (6)	0.3414 (4)	1.22287 (18)	0.0376 (10)*	
N2	0.2838 (11)	0.6044 (3)	1.10975 (16)	0.0376 (10)*	
N3	0.2866 (9)	0.5248 (3)	1.16716 (16)	0.0376 (10)*	
N4	0.2546 (9)	0.8551 (4)	0.81892 (15)	0.0470 (11)*	
N5	0.3124 (9)	0.9785 (4)	0.8192 (2)	0.0470 (11)*	
N6	0.3644 (8)	1.2937 (4)	0.7777 (3)	0.0470 (11)*	
N7	0.2169 (10)	0.9602 (6)	0.6485 (2)	0.0470 (11)*	
C8	0.3240 (7)	0.3997 (3)	1.04589 (17)	0.0376 (10)*	
C9	0.4339 (7)	0.3493 (4)	1.10531 (19)	0.0376 (10)*	
C10	0.3069 (9)	0.5467 (2)	1.05283 (14)	0.0376 (10)*	
C11	0.1216 (10)	0.3375 (5)	1.0463 (3)	0.0376 (10)*	
C12	0.2985 (13)	0.6283 (2)	0.99214 (15)	0.0394 (12)*	
C13	0.3476 (10)	0.4004 (3)	1.16966 (14)	0.0376 (10)*	
C14	0.2772 (11)	0.5712 (3)	0.92960 (18)	0.0394 (12)*	
C15	0.2935 (14)	0.7636 (3)	0.99683 (16)	0.0394 (12)*	
C16	0.2604 (12)	0.6463 (3)	0.87291 (16)	0.0394 (12)*	
C17	0.2957 (13)	0.8390 (3)	0.93951 (18)	0.0394 (12)*	
C18	0.2734 (13)	0.7806 (3)	0.87704 (15)	0.0394 (12)*	
C19	0.2862 (11)	1.0528 (4)	0.7653 (2)	0.0470 (11)*	
C20	0.3161 (9)	1.1878 (4)	0.7732 (3)	0.0470 (11)*	
C21	0.2562 (7)	1.0050 (6)	0.6992 (2)	0.0470 (11)*	
H22	0.39740	0.37220	1.00210	0.0451*	
H23	0.58470	0.38100	1.10290	0.0451*	
H24	0.42550	0.24610	1.10960	0.0451*	
H25	0.05530	0.32640	1.09300	0.0451*	
H26	0.01400	0.39720	1.01670	0.0451*	
H27	0.12240	0.24650	1.01800	0.0451*	
H28	0.24490	0.58260	1.20820	0.0451*	
H29	0.28830	0.46680	0.92150	0.0473*	
H30	0.32240	0.81360	1.04360	0.0473*	
H31	0.28490	0.60190	0.82380	0.0473*	
H32	0.32520	0.94610	0.94320	0.0473*	
H33	0.31980	0.81670	0.77660	0.0564*	

### 2-({4-[(4R)-4-methyl-6-oxo-4,5-dihydro-1H-pyridazin-3-yl]phenyl}hydrazinylidene)propanedinitrile (levosimendan) Geometric parameters (Å, º)

**Table:**

O1—C13	1.227 (3)	C14—C12	1.389 (2)
N2—N3	1.410 (3)	C14—C16	1.376 (2)
N2—C10	1.2931 (13)	C14—H29	1.089 (3)
N3—N2	1.410 (3)	C15—C12	1.396 (2)
N3—C13	1.348 (2)	C15—C17	1.384 (2)
N3—H28	1.053 (2)	C15—H30	1.086 (3)
N4—N5	1.331 (4)	C16—C14	1.376 (2)
N4—C18	1.398 (2)	C16—C18	1.388 (2)
N4—H33	1.036 (5)	C16—H31	1.096 (3)
N5—N4	1.331 (4)	C17—C15	1.384 (2)
N5—C19	1.333 (3)	C17—C18	1.394 (2)
N6—C20	1.143 (3)	C17—H32	1.123 (3)
N7—C21	1.146 (3)	C18—N4	1.398 (2)
C8—C9	1.501 (4)	C18—C16	1.388 (2)
C8—C10	1.5239 (19)	C18—C17	1.394 (2)
C8—C11	1.533 (4)	C19—N5	1.333 (3)
C8—H22	1.050 (4)	C19—C20	1.414 (3)
C9—C8	1.501 (4)	C19—C21	1.424 (3)
C9—C13	1.512 (2)	C20—N6	1.143 (3)
C9—H23	1.089 (6)	C20—C19	1.414 (3)
C9—H24	1.067 (5)	C21—N7	1.146 (3)
C10—N2	1.2931 (13)	C21—C19	1.424 (3)
C10—C8	1.5239 (19)	H22—C8	1.050 (4)
C10—C12	1.4765 (18)	H23—C9	1.089 (6)
C11—C8	1.533 (4)	H24—C9	1.067 (5)
C11—H25	1.046 (7)	H25—C11	1.046 (7)
C11—H26	1.129 (6)	H26—C11	1.129 (6)
C11—H27	1.093 (6)	H27—C11	1.093 (6)
C12—C10	1.4765 (18)	H28—N3	1.053 (2)
C12—C14	1.389 (2)	H29—C14	1.089 (3)
C12—C15	1.396 (2)	H30—C15	1.086 (3)
C13—O1	1.227 (3)	H31—C16	1.096 (3)
C13—N3	1.348 (2)	H32—C17	1.123 (3)
C13—C9	1.512 (2)	H33—N4	1.036 (5)
			
N3—N2—C10	116.61 (13)	H26—C11—H27	101.5 (5)
N2—N3—C13	125.87 (14)	C10—C12—C14	120.21 (13)
N2—N3—H28	107.6 (3)	C10—C12—C15	120.85 (13)
C13—N3—H28	126.3 (3)	C14—C12—C15	118.67 (14)
N5—N4—C18	119.54 (17)	O1—C13—N3	121.45 (18)
N5—N4—H33	103.7 (5)	O1—C13—C9	122.7 (2)
C18—N4—H33	115.4 (5)	N3—C13—C9	114.94 (16)
N4—N5—C19	120.3 (3)	C12—C14—C16	120.84 (13)
C9—C8—C10	108.08 (16)	C12—C14—H29	122.9 (3)
C9—C8—C11	108.0 (3)	C16—C14—H29	115.9 (3)
C10—C8—C11	110.1 (2)	C12—C15—C17	120.23 (17)
C9—C8—H22	108.9 (3)	C12—C15—H30	121.8 (3)
C10—C8—H22	112.4 (3)	C17—C15—H30	116.4 (3)
C11—C8—H22	109.2 (4)	C14—C16—C18	120.31 (14)
C8—C9—C13	110.80 (17)	C14—C16—H31	119.4 (3)
C8—C9—H23	110.0 (4)	C18—C16—H31	117.3 (3)
C13—C9—H23	107.9 (4)	C15—C17—C18	119.91 (15)
C8—C9—H24	112.4 (4)	C15—C17—H32	119.9 (3)
C13—C9—H24	104.9 (3)	C18—C17—H32	120.1 (3)
H23—C9—H24	110.7 (3)	N4—C18—C16	119.42 (19)
N2—C10—C8	123.04 (13)	N4—C18—C17	121.19 (19)
N2—C10—C12	117.21 (11)	C16—C18—C17	119.32 (13)
C8—C10—C12	119.53 (14)	N5—C19—C20	117.0 (3)
C8—C11—H25	116.5 (5)	N5—C19—C21	124.8 (4)
C8—C11—H26	111.5 (4)	C20—C19—C21	117.6 (3)
H25—C11—H26	103.9 (6)	N6—C20—C19	171.3 (8)
C8—C11—H27	110.5 (5)	N7—C21—C19	173.4 (7)
H25—C11—H27	111.8 (5)		

### (poly_caprolactone) Crystal data

**Table:**

C_7_H_12_O_3_	*c* = 17.297 Å
*M**_r_* = 144.17	*V* = 644.92 Å^3^
Orthorhombic, *P*2_1_2_1_2_1_	*Z* = 4
*a* = 7.496 Å	*D*_x_ = 1.485 Mg m^−^^3^
*b* = 4.974 Å	*T* = 295 K

### (poly_caprolactone) Refinement

**Table:**

Weighting scheme based on measured s.u.'s	Preferred orientation correction: March-Dollase correction coef. = 1.000 axis = [0, 0, 1]

### (poly_caprolactone) Fractional atomic coordinates and isotropic or equivalent isotropic displacement parameters (Å^2^)

**Table:**

	*x*	*y*	*z*	*U*_iso_*/*U*_eq_	
O1	0.20480	0.03280	0.05040	0.0300*	
C1	0.29980	−0.03650	0.11820	0.0300*	
H1	0.31360	−0.26400	0.11820	0.0300*	
H2	0.42090	0.09890	0.11820	0.0300*	
C2	0.20030	0.03650	0.19120	0.0300*	
H3	0.19650	0.26380	0.19120	0.0300*	
H4	0.07920	−0.09000	0.19120	0.0300*	
C3	0.29980	−0.03650	0.26440	0.0300*	
H5	0.31360	−0.26400	0.26440	0.0300*	
H6	0.42090	0.09890	0.26440	0.0300*	
C4	0.20030	0.03650	0.33740	0.0300*	
H7	0.19650	0.26390	0.33740	0.0300*	
H8	0.07920	−0.09000	0.33740	0.0300*	
C5	0.29980	−0.03650	0.41060	0.0300*	
H9	0.31360	−0.26400	0.41060	0.0300*	
H10	0.42090	0.09890	0.41060	0.0300*	
C6	0.20030	0.03650	0.48360	0.0300*	
O2	0.05230	0.14480	0.48360	0.0300*	
O1A	0.29520	−0.03280	0.55040	0.0300*	
C1A	0.20020	0.03650	0.61820	0.0300*	
H1A	0.18640	0.26400	0.61820	0.0300*	
H2A	0.07910	−0.09890	0.61820	0.0300*	

### (levosimendan_VASP) Crystal data

**Table:**

C_14_H_12_N_6_O	*b* = 10.29094 Å
*M**_r_* = 280.29	*c* = 19.98971 Å
Orthorhombic, *P*2_1_2_1_2_1_	*V* = 1415.54 Å^3^
*a* = 6.88115 Å	*Z* = 4

### (levosimendan_VASP) Data collection

**Table:**

VASP calculation	*k* = →
*h* = →	*l* = →

### (levosimendan_VASP) Fractional atomic coordinates and isotropic or equivalent isotropic displacement parameters (Å^2^)

**Table:**

	*x*	*y*	*z*	*B*_iso_*/*B*_eq_	
O1	0.34351	0.33805	0.22507		
N2	0.28900	0.60145	0.11095		
N3	0.28810	0.52338	0.16675		
N4	0.27168	0.85295	−0.18238		
N5	0.29933	0.97759	−0.18229		
N6	0.34215	1.29512	−0.22840		
N7	0.22055	0.96110	−0.35824		
C8	0.31576	0.39820	0.04687		
C9	0.42698	0.34320	0.10668		
C10	0.30419	0.54453	0.05297		
C11	0.11116	0.33915	0.04198		
C12	0.29947	0.62633	−0.00706		
C13	0.34862	0.39764	0.17079		
C14	0.28420	0.56979	−0.07082		
C15	0.30634	0.76323	−0.00282		
C16	0.27334	0.64546	−0.12807		
C17	0.29846	0.83982	−0.05964		
C18	0.28114	0.78090	−0.12276		
C19	0.29182	1.04651	−0.23869		
C20	0.32028	1.18248	−0.23230		
C21	0.25313	0.99842	−0.30401		
H22	0.39645	0.37319	0.00131		
H23	0.58198	0.37094	0.10442		
H24	0.41902	0.23681	0.10891		
H25	0.02325	0.36380	0.08617		
H26	0.03436	0.37420	−0.00270		
H27	0.12325	0.23276	0.03974		
H28	0.24712	0.57130	0.20980		
H29	0.27870	0.46472	−0.07700		
H30	0.31818	0.80872	0.04627		
H31	0.25751	0.60048	−0.17707		
H32	0.30544	0.94518	−0.05562		
H33	0.24029	0.80072	−0.22589		

### (levosimendan_VASP) Hydrogen-bond geometry (Å, º)

**Table:**

*D*—H···*A*	*D*—H	H···*A*	*D*···*A*	*D*—H···*A*
N4—H33···O1	1.045	1.826	2.813	156.1
N4—H33···C21	1.045	2.566	2.858	95.2
N3—H28···N6	1.031	1.948	2.947	162.3
C16—H31···O1	1.089	2.170	3.049	136.0
C15—H30···N2	1.090	2.502	2.821	95.3
C9—H24···N7	1.097	2.346	3.366	153.9

## References

[bb1] Altomare, A., Cuocci, C., Giacovazzo, C., Moliterni, A., Rizzi, R., Corriero, N. & Falcicchio, A. (2013). *J. Appl. Cryst.* **46**, 1231–1235.

[bb2] Antao, S. M., Hassan, I., Wang, J., Lee, P. L. & Toby, B. H. (2008). *Can. Mineral.* **46**, 1501–1509.

[bb3] Backman, M., Larma, I., Antila, S. & Lehtonen, L. (2002). United States Patent US 6,355,269 B1.

[bb4] Bernstein, J., Davis, R. E., Shimoni, L. & Chang, N. L. (1995). *Angew. Chem. Int. Ed. Engl.* **34**, 1555–1573.

[bb5] Bittiger, H., Marchessault, R. H. & Niegisch, W. D. (1970). *Acta Cryst.* B**26**, 1923–1927.

[bb6] Bravais, A. (1866). *Etudes Cristallographiques.* Paris: Gauthier Villars.

[bb7] Bruno, I. J., Cole, J. C., Kessler, M., Luo, J., Motherwell, W. D. S., Purkis, L. H., Smith, B. R., Taylor, R., Cooper, R. I., Harris, S. E. & Orpen, A. G. (2004). *J. Chem. Inf. Comput. Sci.* **44**, 2133–2144. 10.1021/ci049780b 15554684

[bb8] Crystal Impact (2025). *DIAMOND V. 5.0.0*. Crystal Impact, Bonn, Germany.

[bb9] Dassault Systèmes. (2025). *BIOVIA Materials Studio 2026*. BIOVIA, San Diego, CA.

[bb10] Donnay, J. D. H. & Harker, D. (1937). *Am. Mineral.* **22**, 446-467.

[bb36] Dovesi, R., Erba, A., Orlando, R., Zicovich-Wilson, C. M., Civalleri, B., Maschio, L., Rérat, M., Casassa, S., Baima, J., Salustro, S. & Kirtman, B. (2018). *WIREs Comput. Mol. Sci.* **8**, e1360.

[bb11] Erba, A., Desmarais, J. K., Casassa, S., Civalleri, B., Donà, L., Bush, I. J., Searle, B., Maschio, L., Edith-Daga, L., Cossard, A., Ribaldone, C., Ascrizzi, E., Marana, N. L., Flament, J.-P. & Kirtman, B. (2023). *J. Chem. Theory Comput.* **19**, 6891–6932. 10.1021/acs.jctc.2c00958 PMC10601489 36502394

[bb12] Etter, M. C. (1990). *Acc. Chem. Res.* **23**, 120–126.

[bb13] Friedel, G. (1907). *Bull. Soc. Fr. Minéral.* **30**, 326-455.

[bb14] Gatti, C., Saunders, V. R. & Roetti, C. (1994). *J. Chem. Phys.* **101**, 10686–10696.

[bb15] Groom, C. R., Bruno, I. J., Lightfoot, M. P. & Ward, S. C. (2016). *Acta Cryst.* B**72**, 171–179. 10.1107/S2052520616003954 PMC4822653 27048719

[bb16] Hirshfeld, F. L. (1977). *Theor. Chim. Acta* **44**, 129–138.

[bb17] Kabekkodu, S., Dosen, A. & Blanton, T. N. (2024). *Powder Diffr.* **39**, 47–59.

[bb18] Kaduk, J. A., Crowder, C. E., Zhong, K., Fawcett, T. G. & Suchomel, M. R. (2014). *Powder Diffr.* **29**, 269–273.

[bb19] Kim, S., Chen, J., Cheng, T., Gindulyte, A., He, J., He, S., Li, Q., Shoemaker, B. A., Thiessen, P. A., Yu, B., Zaslavsky, L., Zhang, J. & Bolton, E. E. (2023). *Nucleic Acids Res.* **51**, D1373–D1380. 10.1093/nar/gkac956 PMC9825602 36305812

[bb20] Kresse, G. & Furthmüller, J. (1996). *Comput. Mater. Sci.* **6**, 15–50.

[bb21] Lee, P. L., Shu, D., Ramanathan, M., Preissner, C., Wang, J., Beno, M. A., Von Dreele, R. B., Ribaud, L., Kurtz, C., Antao, S. M., Jiao, X. & Toby, B. H. (2008). *J. Synchrotron Rad.* **15**, 427–432. 10.1107/S0909049508018438 18728312

[bb22] Macrae, C. F., Sovago, I., Cottrell, S. J., Galek, P. T. A., McCabe, P., Pidcock, E., Platings, M., Shields, G. P., Stevens, J. S., Towler, M. & Wood, P. A. (2020). *J. Appl. Cryst.* **53**, 226–235. 10.1107/S1600576719014092 PMC6998782 32047413

[bb23] Materials Design. (2024). *MedeA 3.7.2*. Materials Design Inc., San Diego, USA.

[bb24] MDI. (2025). *JADE Pro version 9.3*. Materials Data, Livermore, USA.

[bb25] Motherwell, W. D. S., Shields, G. P. & Allen, F. H. (2000). *Acta Cryst.* B**56**, 857–871. 10.1107/S0108768100007230 11006562

[bb26] Silk Scientific. (2013). *UN-SCAN-IT 7.0*. Orem, UT, Silk Scientific Corporation.

[bb27] Singhania, H. (2017). European Patent Application EP 3,424,908 A1.

[bb28] Spackman, P. R., Turner, M. J., McKinnon, J. J., Wolff, S. K., Grimwood, D. J., Jayatilaka, D. & Spackman, M. A. (2021). *J. Appl. Cryst.* **54**, 1006–1011. 10.1107/S1600576721002910 PMC8202033 34188619

[bb29] Stephens, P. W. (1999). *J. Appl. Cryst.* **32**, 281–289.

[bb32] Streek, J. van de & Neumann, M. A. (2014). *Acta Cryst. B* **70**, 1020–1032. 10.1107/S2052520614022902 PMC4468513 25449625

[bb30] Sykes, R. A., McCabe, P., Allen, F. H., Battle, G. M., Bruno, I. J. & Wood, P. A. (2011). *J. Appl. Cryst.* **44**, 882–886. 10.1107/S0021889811014622 PMC3246811 22477784

[bb31] Toby, B. H. & Von Dreele, R. B. (2013). *J. Appl. Cryst.* **46**, 544–549.

[bb33] Wang, J., Toby, B. H., Lee, P. L., Ribaud, L., Antao, S. M., Kurtz, C., Ramanathan, M., Von Dreele, R. B. & Beno, M. A. (2008). *Rev. Sci. Instrum.* **79**, 085105. 10.1063/1.2969260 19044378

[bb34] Wavefunction, Inc. (2025). *Spartan ’24. V. 1.3.1.* Wavefunction Inc., Irvine, USA.

[bb35] Wheatley, A. M. & Kaduk, J. A. (2019). *Powder Diffr.* **34**, 35–43.

